# Association between VTE and antibiotic prophylaxis guideline compliance and patient-reported outcomes after total hip and knee arthroplasty: an observational study

**DOI:** 10.1186/s41687-022-00502-6

**Published:** 2022-10-12

**Authors:** Helen Badge, Tim Churches, Justine M. Naylor, Wei Xuan, Elizabeth Armstrong, Leeanne Gray, John Fletcher, Iain Gosbell, Chung-Wei Christine Lin, Ian A. Harris

**Affiliations:** 1 Whitlam Orthopaedic Research Centre, 1 Campbell Street, Liverpool, 2071 Australia; 2 South Western Sydney Clinical School, UNSW, 1 Elizabeth Street, Liverpool, 2071 Australia; 3 Ingham Institute for Applied Medical Research, 1 Campbell Street, Liverpool, 2071 Australia; 4 Australian Catholic University, 8-20 Napier Street, North Sydney, 2060 Australia; 5 School of Population Health, The University of New South Wales, High St Kensington, Kensington, NSW 2052 Australia; 6 South Western Sydney Local Health District, 1 Elizabeth Street, Liverpool, 2071 Australia; 7 University of Sydney, Fisher Road, Camperdown, NSW 2006 Australia; 8 Westmead Hospital, Cnr Hawkesbury Road and Darcy Road, Westmead, NSW 2145 Australia; 9 Western Sydney University, Campbelltown, NSW 2560 Australia; 10 Sydney School of Public Health, The University of Sydney, Edward Ford Building (A27) Fisher Road, Camperdown, NSW 2006 Australia

**Keywords:** Patient-reported outcome measures, Total knee arthroplasty, Total hip arthroplasty, Complications, Prevention, Surgical site infection, Venous thromboembolism

## Abstract

**Background:**

Surgical site infection (SSI) and venous thromboembolism (VTE) are associated with high burden and cost and are considered largely preventable following total knee or hip arthroplasty (TKA, THA). The risk of developing VTE and SSI is reduced when prophylaxis is compliant with evidence-based clinical guidelines. However, the association between VTE and antibiotic prophylaxis clinical guideline compliance and patient-reported outcome measures (PROMs) after THA/TKA is unknown. This study aims to explore whether care that is non-compliant with VTE and antibiotic guideline recommendations is associated with PROMs (Oxford Hip/Knee Score and EQ-5D Index scores) at 90- and 365-days after surgery.

**Methods:**

This prospective observational study included high-volume arthroplasty public and private sites and consenting eligible participants undergoing elective primary THA/TKA. We conducted multiple linear regression and linear mixed-effects modelling to explore the associations between non-compliance with VTE and antibiotic guidelines, and PROMs**.**

**Results:**

The sample included 1838 participants. Compliance with VTE and antibiotic guidelines was 35% and 13.2% respectively. In adjusted modelling, non-compliance with VTE guidelines was not associated with 90-day Oxford score (β = − 0.54, standard error [SE] = 0.34, p = 0.112) but was significantly associated with lower (worse) 365-day Oxford score (β = − 0.76, SE = 0.29, p = 0.009), lower EQ-5D Index scores at 90- (β = − 0.02 SE = 0.008, p = 0.011) and 365-days (β = − 0.03, SE = 0.008, p = 0.002).. The changes in Oxford and EQ-5D Index scores were not clinically important. Noncompliance with antibiotic guidelines was not associated with either PROM at 90- (Oxford: β = − 0.45, standard error [SE] = 0.47, p = 0.341; EQ-5D: β = − 0.001, SE = 0.011, p = 0.891) or 365-days (Oxford score: β = − 0.06, SE = 0.41, p = 0.880 EQ-5D: β = − 0.010, SE = 0.012, p = 0.383). Results were consistent when complications were included in the model and in linear mixed-effects modelling with the insurance sector as a random effect.

**Conclusions:**

Non-compliance with VTE prophylaxis guidelines, but not antibiotic guidelines, is associated with statistically significant but not clinically meaningful differences in Oxford scores and EQ-5D Index scores at 365 days.

**Supplementary Information:**

The online version contains supplementary material available at 10.1186/s41687-022-00502-6.

## Plain English Summary

Guidelines exist to advise surgeons on how to prevent blood clots and infections for people having total hip and knee joint replacements (arthroplasty surgery). Adhering to these guidelines is associated with a reduced chance of people experiencing blood clots and infections. However, it is not known if guideline compliance affects outcomes reported by the person, including changes in pain, functioning, mobility, and quality of life. This study aimed to determine whether care that adheres to guidelines to prevent blood clots and infection improves the pain, function and quality of life outcomes reported by the person. We assessed the relationship between compliance to these guidelines and outcomes reported by patients in 1838 patients undergoing primary elective hip or knee joint replacement in Australia. When care does not adhere to guidelines to prevent blood clots, the scores on measures of pain and disability due to the operated joint and general quality of life are slightly, but not meaningfully, lower. Care that adheres to guidelines to prevent infection does not affect outcomes reported by the person.

**Supplementary Information:**

The online version contains supplementary material available at 10.1186/s41687-022-00502-6.

## Background

The primary goals of total hip arthroplasty (THA) and total knee arthroplasty (TKA) are to reduce the pain and disability associated with osteoarthritis [1–4]. Understanding the patient's perspective is essential to ensure the objectives of surgery have been met and deliver high-value care in THA/TKA [1, 5, 6]. Patient-reported outcome measures (PROMs) collect information from a patient's perspective and supplement other outcome measures, including implant survival, complications, and cost [7, 8]. The joint-specific Oxford Hip Score (OHS) [9], Oxford Knee Score (OKS) [10] and generic EQ-5D [11] have demonstrated that the majority of people make substantial gains after THA and TKA, including reduced pain and improved function and quality of life [3, 12, 13].

Quality of life outcomes are associated with age, pre-operative pain, functional levels [14], patient expectations [15], psychosocial factors and the experience of complications [16–18]. Complications including venous thromboembolism (VTE) and surgical site infection (SSI) are associated with reduced quality of life, increased burden, and costs [19–21]. Compliance with clinical guidelines to prevent complications such as VTE and SSI is associated with a reduced risk of experiencing complications [22, 23]. However, the association between non-compliant care and patient-reported outcomes is unknown. This study aims to determine if non-compliance with VTE and antibiotic clinical guideline recommendations are associated with patient-reported outcomes (Oxford score and EQ-5D Index score) up to 365 days after THA or TKA.

## Methods

The study was a prospective observational study including high-volume arthroplasty sites and consenting eligible participants undergoing elective primary THA/TKA for osteoarthritis. Public and private high volume (> 275 per annum) joint replacement centres in Australia were recruited through random and convenience sampling. Participants were eligible if they were adults aged over 18 years, undergoing primary THA or TKA, spoke sufficient English and who reported that they were available to participate in telephone interviews up to 365 days following surgery. Ethics approvals were obtained from nine committees, and the protocol was registered with clinicaltrials.gov (Identifier NCT01899443) before the study commenced [24]. High-volume (> 275 cases annually) were recruited through random and then convenience sampling to improve the slower than expected recruitment rates. Consecutive eligible participants were recruited by the site coordinator during the usual process for preadmission assessment at each site, either in clinic or via telephone.

The criteria for compliance were developed by a panel including orthopaedic surgeon, nurse unit manager, haematologist, infectious diseases physician, physiotherapist researcher, two biostatisticians, and the Arthroplasty Clinical outcomes Registry National (ACORN) [25] manager. The research team used an iterative consensus process to agree on precise criteria for compliance versus non-compliance with each care element within the guideline recommendations (Additional file 1).

Participants reported baseline data, including demographics, information about their osteoarthritis, previous THA or TKA, medications for pain, comorbid health conditions, and patient-reported measures before surgery. Baseline data were collected at the time of consent or within a week of consent. Sites provided data regarding the surgical procedure, anaesthesia, acute care, antibiotic and VTE prophylaxis and any complications during the acute admission. Surgical complications included all-cause mortality and VTE up to 365 days, joint-related readmissions and reoperations up to 365 days, and non-joint related hospital readmissions and reoperations up to 35 days. Follow-up data were collected via telephone at 35, 90 and 365 days and included details about post-acute complications, VTE prophylaxis, health service utilisation and follow-up PROMs. The PROMS were completed based on the person’s perceptions on the day of collection. The EQ5D asks for people to rate their perceived health on that day. The Oxford Hip and Knee scores asks the person to report their perceived status for the preceding four weeks. Sites and participants reported complications. The research team verified these through contacting sites, GPs, surgeons, and an audit of medical records.

Compliance was calculated with the recommendations of two nationally produced guidelines, that were considered to be the most commonly used and important guidelines for Australian health services:

I. National Health and Medical Research Council (NHMRC) Clinical Practice Guideline for the Prevention of Venous Thromboembolism (Deep Vein Thrombosis and Pulmonary Embolism) in Patients admitted to Australian hospitals (2009) [26]; and
II. Therapeutic Guidelines (TG) Antibiotic Version 14 (2010) [27]

Compliance was assessed as a series of dichotomous variables for each of the four elements of compliance for both the VTE and antibiotic guidelines. To be considered compliant, the care needed to meet all criteria for each element of that guideline. Determining compliance required assessing complex data regarding each person's prophylaxis and patient-specific indications and contra-indications. Compliance results were automatically calculated in the R language for statistical reporting and programming based on data regarding participant characteristics and the prophylaxis they received [28] (Additional file 1: Table S1).

## Patient-reported outcome measures

### Oxford scale

The Oxford Hip Score (OHS) [9] and the Oxford Knee Score (OKS)[10] are brief, valid and responsive measures used to measure the impact of TKA and THA on pain and functioning [1, 12, 29, 30]. The Oxford scales were chosen as the key joint-specific patient reported outcome measures as these are used in the Australian and international joint replacement registries and there is strong evidence supporting their psychometric properties and clinical utility [1, 12, 29, 30]. Both the OHS and OKS have twelve items scored on a five-point Likert scale with total scores ranging from 0 to 48, with higher scores representing better functioning [9, 10]. Using anchor-based methods, the minimally important change (MIC) [31] that represents the change score associated with minimally improved function is ten to eleven points for the OHS [32, 33] and eight to nine points for the OKS [32, 34]. The minimally important difference (MID) score for between-group comparison is five points for both measures [32]. The total score value indicating the success of surgery is 32.5 to 38.5 for OHS and 28.5 to 36.5 for OKS [35]. To evaluate outcomes from THA and TKA the optimal time for follow-up is six to 12 months following surgery [36]. Administration of the Oxford Scores has been validated via face-to-face interviews, self-administration and telephone [37]. The research team administered the Oxford Hip or Knee score via face-to-face interview for baseline assessments and telephone interview with the participant post-operatively at 90 and 365 days [9, 10].

### EQ-5D

The EQ-5D is one of the most frequently used generic measures of patient-reported outcomes evaluating perceived health-related quality of life with patients undergoing total joint arthroplasty [11, 38, 39]. The EQ-5D-5L comprises five individual domains measuring the extent of problems experienced on that day due to any health condition (self-care, usual activities, pain/discomfort, and anxiety/depression) rated using a five-point Likert scale. The person also scores their overall quality of life on that day on a zero to 100 visual analogue scale (VAS), reflecting the worst to best health they can imagine [9]. The research team administered the EQ-5D via face-to-face interview for baseline assessments and telephone interview with the participant post-operatively at baseline, 35, 90 and 365 days [11, 40].The EQ-5D domain scores for the four data collection points were entered into an online tool to calculate a single health state index score for each point in time to use in data analyses [9]. The Canadian value set for EQ-5D was used as Australian value sets for EQ-5D have not been published and based on the assumption that the Australian population may have similar preferences [9, 33, 36]. The EQ-5D is responsive to measure change after THA and TKA [41, 42], with MIC scores 0.20 to 0.41 for THA and 0.22 for TKA [33].

### Data analyses

All analyses were conducted using the R Statistical Computing Platform (version 3.6.1) [28]. Descriptive statistics were calculated to profile site-level and participant-level characteristics. Results were presented as median and inter-quartile range (IQR) or mean and standard deviation (SD). Some variables (bilateral joint, smoking status, five-point American Society of Anesthesiology score [ASA] [43], education, neuraxial anaesthesia) were collapsed to allow for adequate sample size or clinically meaningful groups to be included in analyses. Unadjusted (bivariable) analyses were undertaken for each outcome. Differences in PROMS were described using t-tests. The difference in scores for each PROM were calculated between the baseline and 365-day follow-up interview. These results were used to determine how many participants exceeded the MIC for both OHS/OKS and EQ-5D. This is a secondary analysis of data, and the sample size was based on the expected event rates and effect size of the primary study [24].

We conducted a series of adjusted multiple linear regression analyses to explore the associations between binary system-level non-compliance with VTE guidelines and antibiotic guidelines with the EQ5D and Oxford scores, with separate analyses for 90-day and 365-day PROMs. For each timepoint, the Oxford Hip and Knee scores were merged into a single “Oxford score’ variable, with joint included to differentiate them in modelling. Patient, surgical, and care factors provided by participants and sites (Table 1) were considered potential confounders for all adjusted models [44–46]. Factors identified on unadjusted analysis with a p-value < 0.25 were entered into a backwards, stepwise multivariable linear regression model or multivariable logistic regression model (using the Akaike information criterion—AIC) to identify the association between guidelines compliance and PROMs as relevant for each analysis (Additional file 1). The main predictors (non-compliance) and baseline PROMs were forced into the model, the latter to adjust for differences in pre-operative functioning. Missing data were imputed using multivariate imputation by chained equations (MICE). Model selection was performed using one of the imputed datasets. Effect estimates were taken from the pooled estimates using the five imputed datasets. Interaction terms for the main predictor (non-compliance) against each other variable were tested in the final model for each analysis.

**Table 1:** Site, surgeon, participant and care characteristics

	Description,	Results N (%), median (IRQ)
*Site & surgeon characteristics*		
Sites	Public	10 (54%)
	Private	9 (46%)
Number surgeons		118
Number participants	Per surgeon [(Median (IQR))	58 (31, 101)
	Per site (Median (IQR))	70 (42.5, 127)
Length of stay (days)	Median (IQR)	5 (1.22, 1.95)
*Participant characteristics*		
Joint (all surgeries)	Hip	801 (43.6%)
	Knee	1037 (56.4%)
Bilateral joint arthroplasty		91 5.0%)
Public hospital	Yes	841 (45.8%)
Duration of surgery (hours) (N = 1837)	Median (IQR)	1.6 (1.2, 1.0)
Length of stay (s = days)	Median (IQR)	5.0(4.0, 7.0)
Age (years)	Median (IQR)	67.6 (61.0, 73.9)
Sex	Female	1001 (54.5%)
Insurance status	Public	801 (43.6%)
	Private health insurance	965 (52.5%)
	Self-funded (private)	29 (1.6%)
	Other insurance / compensation	16 (0.9%)
	Department of Veterans Affairs	27 (1.5%)
Post-school education status (N = 1866)	Up to school completion	874 (45.5%)
	Post school qualification	958 (54.5%)
BMI	Median (IQR)	29.7 (26.3, 34.2)
Current smoker (N = 1828)	Yes	150 (8.2%)
Comorbid conditions	Heart disease	459 (25.0%)
	History stroke	111 (6.0%)
	Bleeding disorder	19 (1.0%)
	Previous VTE (N = 1873)	146 (8.0%)
	Diabetes	298 (16.2%)
	Hypertension	1118 (60.8%)
	High cholesterol	685 (37.3%)
	Kidney disease	56 (3.0%)
	Liver disease	46 (2.7%)
	Current cancer (any type)	37 (2.0%)
	History of any type of cancer (N = 1873)	214 (11.7%)
	Respiratory disease	333 (18.1%)
	Anxiety or depression	342 (18.6%)
	Mental health disorder	22 (1.2%)
	Gastro-intestinal Reflux Disorder (GORD)	473 (25.7%)
	Sleep apnoea	129 (7.0%)
	Neurological conditions	51 (2.8%)
	Any other musculoskeletal condition (N = 1873)	886 (48.2%)
	Any other comorbid conditions not yet specified	712 (38.7%)
Previous total joint arthroplasty	Hip	238 (12.9%)
	Knee	301 (16.4%)
Medications taken for Osteoarthritis*	Any	1438 (78.3%)
	Paracetamol	1064 (57.9%)
	Non-steroidal anti-inflammatories (NSAIDS)	511 (27.8%)
	Opioids	378 (20.6%)
	Antidepressant / antiepileptics, e.g. amitriptyline	35 (1.9%)
	Steroids	5 (0.3%)
Any indications or contraindication for antibiotics		249 (13.7%)
	History of antibiotic resistant infection or swab	
	MRSA	83 (4.5%)
	Gram negative infection	1 (0.05%)
	Self-reported allergy to penicillin, cephalosporin or all beta-lactam Abs	220 (12.0%)
	Hospital admission with LOS > 5 days within three months of THA or TKA	13 (0.7%)
American Association Anaesthetists (ASA) score (N = 1798)	1 or 2	1225 (68.1%)
	3 or 4	573 (31.9%)
Acute processes of care	Routine doppler performed (N = 1847)	146 (8.0%)
	Cement fixation used (N = 1837)	1178 (64.1%)
	Antibiotic cement	1117 (61.0%)
	Tranexamic acid used (N = 1831)	1105 (60.3%)
	Neuraxial anaesthesia (N = 1837)	1163 (63.3%)
	Intra-articular Drain (N = 18)	803 (43.8%)
	Tourniquet (only used for TKA)	886 (48.2%)
	Blood transfusion (N = 1831)	326 (17.8%)
	Indwelling catheter	1435 (78.1%)
Antibiotic prophylaxis	Cephazolin	1668 (90.8%)
	Flucloxacillin	82 (4.5%)
	Vancomycin	133 (7.2%)
	Other cephalosporins (excluding cephazolin)	236 (12.8%)

VTE Prophylaxis	Description	
Mechanical prophylaxis	SCD, calf compressors / foot pumps (N = 1810)	1657 (90.2%)
	Graduated compression stockings (GCS) (N = 1835)	1385 (76.5%)
	Wore any mechanical prophylaxis device (SCD/GCS)	1811 (98.5%)
Duration of any mechanical prophylaxis (days) (N = 1809)	Median (IQR)	27 (15,38)
Mobilisation post-surgery	First mobilised day 0 or 1	1368 (74.8%)

Chemical prophylaxis	Description of preoperative use N (%)	
Low molecular weight heparins (LMWH)	13 (0.7%)	1446 (78.7%)

Care characteristics	Description of preoperative use N (%)	Description of postoperative use N (%)
Aspirin (N = 1836)	458 (24.9%)	861 (46.8%)
Warfarin	66 (3.6%)	75 (4.1%)
Rivaroxaban	13 (0.7%)	158 (86.6%)
Dabigatran etexilate	9 (0.5%)	11 (0.6%)
Unfractionated heparin	4 (0.2%)	73 (4.0%)
Apixaban	2 (0.1%)	10 (0.54%)
Duration of VTE chemical prophylaxis (days)(N = 1831)	Median (IQR)	22 (12, 36)

^*^Medications not exclusive, people may have been taking multiple medications for pain

Sensitivity analyses were performed using complete case analysis, including complications in the final models, and Bayesian information criterion (BIC) instead of AIC for the stepwise regression modelling. Further sensitivity analyses included linear mixed effects modelling for 365-day outcomes to ensure no mediating effects for with and without hospital insurance sector (public or private) as a random effect (it was not possible to explore individual site impacts due to low numbers for some sites). A de-identified version of the data set and the complete R code for all analyses are available (https://doi.org/10.26190/c46r-ne05).

## Results

### Sample ascertainment

**Fig. 1:**
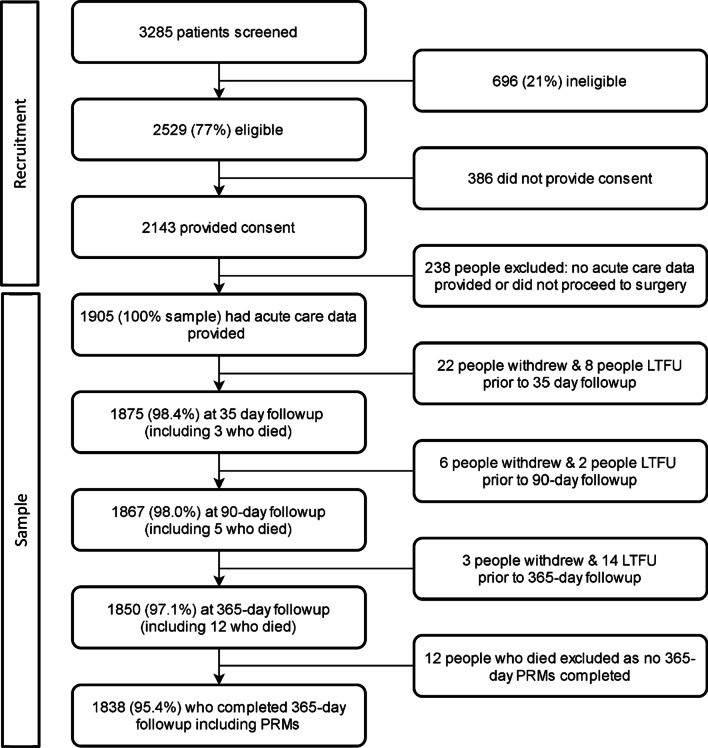
Participant recruitment, eligibility, and participation results

Nine public and ten private sites participated in the study. Seventy-seven percent (2529/3285) of all patients screened were eligible for participation (Fig. 1). Of these, 2143 people provided consent preoperatively, and data were received for 1905 (88.9%) consenting participants as some did not proceed to surgery or no acute data were received by investigators. The sample included 58% (1905/3285) of the potential participants that were screened. The 12 people who died and a further fifty-five (2.9%) people were excluded from analyses as they did not have patient-reported measures collected at 365 days follow up, leaving 1838 participants in this study. Missing data for each variable were less than 2% for all variables except ASA class (2.2% missing).

Sites, Surgeon and Participant Characteristics

The site, surgeon, and participant characteristics are described in Table 1.

Patient-reported outcome measures up to 365 days.

**Fig. 2:**
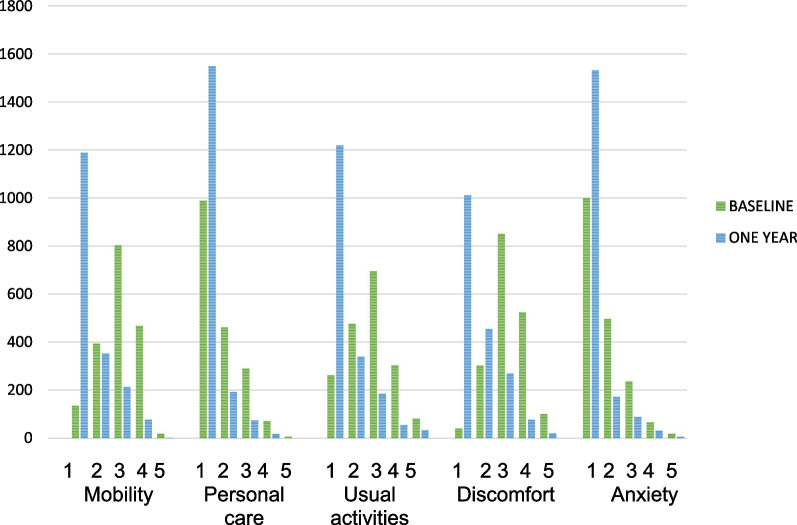
Distribution of EQ5D domain scores at baseline and one year

Table 2 describes the patient-reported outcome measure scores, including Oxford scores, EQ-5D Index and visual analogue scale (VAS) scores at baseline, 90-, and 365-days post-surgery and timeframes for collection. Figure 2 describes the EQ-5D domain scores.

**Table 2:** Patient-reported outcomes (N = 1838)

Patient-reported measures	Baseline	90 days	12 months
Timeframe (days post-surgery) median (IQR)	(N = 1875)	(N = 1862) 91 (90,92)	(N = 1838) 364 (358,366)
Oxford Hip Score (OHS) (N = 786) Median (IQR)	(N = 789) 21 (15, 27)	(N = 795) 45 (40,47)	(N = 792) 47(45, 48)
Change Oxford hip score			25 (18,31)
MIC (change ≥ 11)* NoYes			66 (8.4%) 720 (91.6%)
Oxford Knee Score (OKS) (N = 1028) Median (IQR)	(N = 1030) 21 (15,27)	(N = 1025) 38.5 (33,43)	(N = 1025) 43 (39 45)
Change OKS			20 (14,42)
MIC (change ≥ 9)* NoYes			123 (12.0%)905 (88.0%)
Combined Oxford scores Median (IQR)	(N = 1853) 21 (15, 27)	(N = 1853) 41 (36,45)	(N = 1833) 45 (42, 48)
MID for THA/TKA* NoYes			212 (11.5%)1626 (88.4%)
EQ5D Index score Median (IQR)	(N = 1850) 0.67 (0.44, 0.78)	(N = 1839) 0.81 (0.7, 0.9)	(N = 1854) 0.89 (0.8,1.0)
MIC THA (≥ 0.20)* (N = 790) NoYes			229 (29.1%%)561 (71.0%)
MIC TKA (≥ 0.22)* (N = 1017) NoYes			500 (49.2%)517 (50.1%)
EQ5D VAS score Median (IQR)	(N = 1823) 75 (60, 85)	(N = 1810) 85 (75,90)	(N = 1796) 85 (75, 91)

### Surgical complications

Table 3 describes the surgical complications experienced by participants, including any VTE, any surgical site infection or deep SSI only (requiring IV antibiotics, readmission or reoperation) up to 365-days post-surgery, joint-related readmissions and reoperations up to 365 days post-surgery and non-joint related readmissions and reoperations up to 35-days post-surgery.

**Table 3:** Surgical complications

Type of surgical complications	During acute admission	Between acute discharge and 35 days	Between 36 and 90 days	Between 91 and 365 days	Total N of events*	N of people* N (%)
Any VTE	37 (2.0%)	30 (1.6%)	8 (0.4%)	5 (0.3%)	80 (4.4%)	73 (4.0%)
Readmission	NA	78 (4.2%)	49 (2.7%)	54 (2.9%)	181 (9.8%)	156 (8.5%)
Reoperation	14 (0.8%)	33 (1.8%)	33 (1.8%)	43 (2.3%)	123 (6.7%)	106 (5.8%)
Any SSI	94 (5.1%)	111 (6.0%)	53 (2.9%)	8 (0.4%)	266 (14.5%)	251 (13.7%)
Deep SSI	23 (1.3%)	22 (1.2%)	11 (0.6%)	6 (0.3%)	62 (3.4%)	60 (3.3%)

^*^The difference between total number of events and number of people includes those people who experienced multiple separate events

### VTE and infection prophylaxis and clinical guideline compliance.

The level of compliance with NHMRC VTE clinical guidelines and TG Antibiotic for preventing SSI recommendations are described in Table 4, including care provided by hospitals and self-administered VTE prophylaxis after discharge as relevant.

**Table 4:** VTE and antibiotic clinical guideline compliance

	Yes (N, %)
*Criteria for VTE compliance*	
1. Right drug (N = 1875)	1487 (80.9%)
2. Right dosage (N = 1860)	1297 (71.1%)
3. Right duration (Hip: ≥ 28 days, Knee: ≥ 10 days) (N = 1875)	828 (45.0%)
4. Right mechanical device / joint N = 15	1670 (90.9%)
Compliant with NHMRC VTE clinical guidelines	643 (35.5%)
*Criteria for Antibiotic compliance*	
1. Right drug (N = 1875)	1389 (75.6%)
2. Right dosage (including intra-op dose for op > 3 h) (N = 1874)	437 (23.8%)
3. Right pre-op timing (any) (N = 1875)	1745 (94.9%)
4. Right duration (N = 1875)	1007 (54.8%)
Compliant with TG Antibiotic clinical guideline	243 (13.2%)

### Complication rate by VTE and antibiotic non-compliance.

The rate of VTE, all SSI and deep SSI were higher in both the VTE and antibiotic non-compliant groups compared to those who received compliant prophylaxis (Table 5).

**Table 5:** VTE and SSI complications by VTE and antibiotic compliance and non-compliance

Complications by one year	VTE clinical guidelines (N = 1823)		Therapeutic guidelines Antibiotic (N = 1936)	
	VTE compliance N (%)	VTE non-compliance N (%)	TGAb noncompliance N (%)	TGAB non-compliance N (%)
VTE	16 (0.88%)	57 (3.1%)	6 (0.33%)	67 (3.7%)
All SSI	80 (4.4%)	171 (9.4%)	15 (0.8%)	236 (12.9%)
Deep SSI	20 (1.1%)	40 (2.2%)	3 (0.2%)	57 (3.1%)

### Association between VTE and antibiotic clinical guidelines non-compliance and Oxford score at 90 and 365 days

In unadjusted analyses (t-tests), the difference in mean Oxford scores for people who received prophylaxis that was non-compliant with VTE clinical guidelines compared to those who compared to those who received compliant prophylaxis, were significantly but not clinically important at 90 days (38.9 vs. 39.8, p = 0.011), with no differences at 365 days (42.94 vs. 43.18, p = 0.44). The differences in mean Oxford scores for people who received prophylaxis that was non-compliant with antibiotic clinical guidelines compared to those who received compliant prophylaxis were significant but not clinically important at 90 days (41.04 vs. 39.29, p = 0.0004) and 365 days (44.04 vs. 42.97, p < 0.013).

In adjusted modelling neither non-compliance with VTE clinical guidelines (β = − 0.54, standard error [SE] = 0.33, p = 0.112) or noncompliance antibiotic clinical guidelines (β = − 0.45, standard error [SE] = 0.47, p = 0.341 were associated with the Oxford score at 90 days (Table 6). Variables that met the criteria for inclusion in the initial 90- and 365-day Oxford score models are described in Additional file 1: Table S2 and Additional file 1: Table S3. In adjusted modelling, lower Oxford score at 365-days was associated with non-compliance with VTE clinical guidelines (β = − 0.76, standard error [SE] = 0.29, p = 0.009) (Table 6). Noncompliance with antibiotic guidelines were not associated with the Oxford score at 365 days (β = 0.06, SE = 0.41, p = 0.88). A single interaction term between VTE clinical guidelines non-compliance and taking an antidepressant or anticonvulsant medication for pain such as amitriptyline preoperatively was significant. When people who were taking a pre-operative antidepressant or anticonvulsant medication for pain were removed, the estimates were similar. Non-compliance with VTE clinical guidelines remained significantly associated with Oxford scores at 365 days when missing data were removed, and when complications (readmission, reoperation, VTE, any SSI, deep SSI) were included in the final model (β = − 0.67, SE = 0.31, p = 0.03). In linear mixed effects modelling using the hospital insurance sector (public or private hospital setting) as a random effect, VTE clinical guidelines non-compliance remained significantly associated with Oxford score at 365 days (β = − 0.72, SE = 0.29, p = 0.01).

**Table 6:** Association between antibiotic and VTE non-compliance and 90-day Oxford scores

	Beta Estimate	SE	p value
*Final model for 90-day Oxford scores*			
Non-compliance NHMRC VTE clinical guidelines	− 0.54	0.34	0.112
Non-compliance TG Antibiotic clinical guidelines	− 0.45	0.47	0.341
Oxford Score at baseline	0.12	0.02	< 0.0001
EQ-5D Baseline VAS score	0.03	0.01	0.005
TKA	− 5.77	0.33	< 0.0001
Female sex	− 1.44	0.32	0.00001
Longer surgical duration	− 0.76	0.28	0.007
Comorbid GORD	− 1.10	0.36	0.002
First mobilised day 0 or 1	1.08	0.37	0.004
Comorbid musculoskeletal condition	− 0.91	0.33	0.005
Took a preoperative antidepressant or anticonvulsant medication for pain	− 3.42	1.16	0.003
Took a preoperative opioid medication for pain	− 0.62	0.42	0.13
Current smoker	− 1.15	0.58	0.046
Took a preoperative NSAID for pain	0.60	0.35	0.086
*Final model for 365-day Oxford scores*			
Non-compliance NHMRC VTE clinical guidelines	− 0.76	0.29	0.009
Non-compliance TG Antibiotic clinical guidelines	0.06	0.41	0.880
TKA	− 4.02	0.230	< 0.0001
Female sex	− 0.96	0.28	0.001
Took a preoperative antidepressant or anticonvulsant medication for pain	− 3.13	0.100	0.002
Oxford Score at baseline	0.07	0.02	0.003
Any other comorbid musculoskeletal condition (type not specified)	− 0.83	0.28	0.003
ASA score 3 or 4	− 0.87	0.31	0.006
Comorbid GORD	− 0.71	0.32	0.024
Private hospital	0.69	0.31	0.026
Comorbid sleep apnoea	− 1.09	0.56	0.051
First mobilised day 0 or 1	0.62	0.33	0.060
Current smoker	− 0.85	0.50	0.091
Body mass index (BMI)	− 0.04	0.02	0.095
Received routine doppler ultrasound (acute)	− 0.57	0.38	0.136
EQ-5D baseline Index score	1.14	0.81	0.158

### Association between VTE and antibiotic non-compliance and EQ-5D Index score at 90 and 365 days 

In unadjusted analyses (t-tests), the difference in mean EQ-5D Index scores for people who received prophylaxis that was non-compliant with VTE clinical guidelines compared to those who received compliant prophylaxis were significant but not clinically important at 90 days (0.849 vs. 0.846, p = 0.708) and 365 days (0.881 vs. 0.871, p = 0.253). The difference in mean EQ-5D Index scores for people who received prophylaxis that was non-compliant with antibiotic clinical guidelines compared to those who received compliant prophylaxis were not statistically different at 90 (0.861 vs. 0.845, p = 0.19) and were significant but not clinically important at 365 days (0.898 vs. 0.872, p = 0.022).

In adjusted modelling, lower EQ-5D Index scores at 90 days was associated with non-compliance with VTE clinical guidelines (β = − 0.02, SE = 0.01, p = 0.01), but not non-compliance with antibiotic guidelines (β = − 0.001, SE = 0.01, p = 0.89) (Table 7). A single interaction term between VTE clinical guidelines non-compliance and people with comorbid anxiety or depression was significant. When people with comorbid anxiety or depression were removed from the model, VTE clinical guidelines non-compliance was no longer significant. Variables that met the criteria for inclusion in the initial 90- and 365-day EQ-5D Index score models are described in Additional file 1: Table S4 and Additional file 1: Table S5. In adjusted modelling, factors associated with a lower EQ-5D Index scores at 365 days included non-compliance with VTE clinical guidelines (β = − 0.03, SE = 0.008, p = 0.002) (Table 7). Non-compliance with antibiotic clinical guidelines was not associated with the EQ-5D Index score at 365 days (β = − 0.01, SE = 0.01, p = 0.38). A single interaction term between VTE clinical guidelines non-compliance and people with comorbid anxiety or depression was significant. When people with comorbid anxiety or depression were removed from the model, VTE clinical guidelines non-compliance was no longer significant. Non-compliance with VTE clinical guidelines remained significantly associated with EQ-5D Index scores when missing data were removed. Non-compliance with VTE clinical guidelines (β = 0.02, SE = 0.1, p = 0.007) also remained significantly associated with EQ-5D Index scores at 365 days when complications (readmission, reoperation, VTE, any SSI, deep SSI) were included in the final model; no complications were significantly associated with EQ-5D scores. In linear mixed effects modelling using the hospital insurance sector as a random effect, VTE clinical guidelines non-compliance remained significantly associated with EQ-5D Index scores at 365 days (β = − 0.02, SE = 0.008 p = 0.004).

**Table 7:** Association between clinical guideline non-compliance and EQ-5D Index score at 90 days

	Beta Estimate	SE	p value
*Final model for 90-day EQ-5D Index scores*			
Non-compliance NHMRC VTE clinical guidelines	− 0.02	0.01	0.011
Non-compliance TG Antibiotic clinical guidelines	− 0.001	0.01	0.891
EQ-5D baseline Index score	0.13	0.02	< 0.0001
EQ-5D baseline VAS score	0.001	< 0.001	< 0.0001
TKA	− 0.05	0.01	< 0.0001
Comorbid musculoskeletal condition	− 0.04	0.01	< 0.0001
Took a preoperative opioid medication for pain	− 0.04	0.01	< 0.0001
Comorbid depression or anxiety	− 0.02	0.01	0.019
ASA score 3 or 4	− 0.02	0.01	0.048
Female sex	− 0.02	0.01	0.039
Comorbid depression or anxiety	− 0.07	0.03	0.011
Surgical duration	− 0.01	0.01	0.112
First mobilised day 0 or 1	0.02	0.01	0.051
Comorbid neurological condition	− 0.04	0.02	0.055
Current smoker	− 0.03	0.01	0.043
Age	0.001	< 0.001	0.119
Comorbid liver disease	− 0.03	0.02	0.156
Comorbid current cancer	− 0.04	0.03	0.111
*Final model for 365-day EQ-5D Index scores*			
Non-compliance NHMRC VTE clinical guidelines	− 0.03	0.01	0.002
Non-compliance TG Antibiotic clinical guidelines	− 0.01	0.01	0.383
EQ-5D Baseline Index score	0.11	0.02	< 0.0001
EQ-5D Baseline VAS score	0.001	< 0.001	0.003
Oxford baseline score	0.001	0.001	0.109
TKA	− 0.05	0.01	< 0.0001
Took a preoperative antidepressant or anticonvulsant medication for pain	− 0.07	0.03	0.015
Took a preoperative opioid medication for pain	− 0.04	0.01	< 0.0001
ASA score 3 or 4	− 0.02	0.01	0.011
Bilateral THA/TKA	0.06	0.02	0.001
Comorbid depression / anxiety	− 0.03	0.01	0.002
Comorbid lung disease	− 0.02	0.01	0.046
Comorbid musculoskeletal condition	− 0.04	0.01	< 0.0001
Other comorbid disease (not specified)	− 0.02	0.01	0.065
Comorbid sleep apnoea	− 0.04	0.02	0.004
History of stroke	− 0.03	0.02	0.084
Previous THA	− 0.03	0.01	0.020
Private hospital	0.02	0.01	0.086
Neuraxial anaesthesia	0.02	0.01	0.043
First mobilised day 0 or 1	0.02	0.01	0.012

## Discussion

Non-compliance with VTE clinical guidelines was associated with lower Oxford scores at 365 days and lower EQ-5D Index scores at 90 and 365 days. Based on studies arising from the Australian National Joint Replacement Registry, we anticipated the possibility of variation in the effect between public and private sector hospitals [47]; however, VTE clinical guidelines non-compliance remained significantly associated with Oxford Scores and EQ-5D Index scores in linear mixed-effects modelling with insurance sector as a random effect. The association between VTE clinical guidelines non-compliance and PROM scores may not be clinically meaningful. There was less than one point difference in average 90- and 365-day Oxford scores based on VTE compliance, which is well below all measures of clinically relevant change [32, 34]. The difference in EQ-5D scores was also well below clinically meaningful change for THA [41, 48] and TKA [33]. When complications were included in the models, there was less than a 10% change in the estimates for non-compliance with VTE clinical guidelines for Oxford scores and EQ-5D Index scores at 365 days, suggesting that it is unlikely that the experience of complications mediated these associations.

Patient reported outcome measures have been associated with VTE both before and after surgery. Lower pre-operative Oxford Hip scores are associated with an increased chance of pre-operative VTE [49]. After TKA, poorer Oxford Knee scores and EQ-5D at one year are associated with VTE, suggesting VTE has a small to important impact on patients’ lives [50, 51]. A systematic review indicated that patients put higher value on VTE risk reduction than on the potential harms of the treatment, which may also explain the association we reported between non-compliant VTE prophylaxis and PROMs [50]. The use of different VTE prophylactic agents has varying impacts on patient outcomes, the risk of complications and prophylaxis related adverse events and hospital length of stay [52]. We were interested to explore whether non-compliant VTE and antibiotic prophylaxis were associated with PROM after THA/TKA.

Previous research has demonstrated that treatment adherence is associated with health outcomes, including patient satisfaction [53], quality of life [54], and non-adherence is associated with increased morbidity and costs [55]. Patients often lack clear understanding of their personal risk of VTE and SSI this may contribute to suboptimal levels of compliance with recommended prophylaxis [56, 57]. We did not collect patient reasons for non-adherence, although there is inconsistent evidence that patient preference for mode of administration, dose and duration impact their adherence to VTE prophylaxis [58–60]. We acknowledge that decisions about VTE prophylaxis by both clinicians and patients influence the degree to which VTE prophylaxis is compliant. In contrast, antibiotic prophylaxis is usually finished within 24 h of surgery [27], and the patient is not required to do anything, which may explain the lack of between antibiotic clinical guidelines and PROMs.

Previous research has, demonstrated that non-compliance with clinical guidelines is associated with an increased risk of complications, including VTE and SSI, which in turn are associated with poorer patient-reported outcome measures [22, 51]. In this study, most people who experienced VTE did so in the first 35 days of surgery [61], thus, resolution of VTE signs and symptoms may mean patient -reported recovery measured at 365 days is unaffected by this complication. However, other studies have reported poorer Oxford Knee Scores and EQ-5D at one year are associated with VTE, suggesting VTE has a small to important impact on patients’ lives [50, 51]*.*The association between clinical guidelines non-compliance and complications seems the most likely link to explain the association between VTE non-compliance and poorer outcomes, although the effect persisted when complications were included in the final model. However, this study may be underpowered to detect the mediating effect of complications.

This study is novel as we have explored a direct link between evidence-based guideline recommendations and PROMs rather than looking at complications. While reducing avoidable complications such as VTE and SSI are key targets to improve the value of THA/TKA, value needs to be assessed from multiple perspectives, including the patients [62–64]. The study’s strengths include the rigorous prospective data and the automated non-compliance calculations that accommodated patient-appropriate variations. We used validated measures with proven responsiveness to measure change after knee or hip arthroplasty [12, 65]. In line with EuroQoL recommendations, we used a Canadian value set for EQ-5D assuming that the Australian population may have similar preferences. We could also have used an Australian crosswalk value set from EQ-3D, although this tool has inferior psychometric properties to the EQ-5D [11, 38, 66].

There are several limitations to this study. There are complex personal, surgical, care and system level factors that influence the risk of complications, patient reported outcomes and costs associated with THA/TKA [67–69]. We attempted to account for patient and care factors that may mediate patient-reported measures, but there may be other unmeasured confounders we did not consider or were unable to measure [64]. For example, pre-operative patient expectations or patient activation, whereby compliant people may do other things that influence their outcomes after THA/TKA [15, 70]. Surgeon factors that influence decisions regarding adherence [53, 71], and the surgeon’s volume and surgical proficiency that may have more direct impacts on patient outcomes [72]. Different outcome measures and criteria used to determine compliance may yield different results. More compliant hospitals may also be more rigorous with other processes of care. Further research should explore the differences in quality-adjusted life years (QALYs) and the economic impact of clinical guidelines non-compliance [73].

In conclusion, non-compliance with VTE or antibiotic clinical guidelines does not appear to be associated with patient-reported outcomes following THA or TKA as assessed here. The lack of meaningful association with PROMs does not undermine the importance of providing evidence-based care that reduces the risk of VTE and SSI.

## Supplementary Information

**Additional file 1.** **Supplementary Table 1:** Criteria for compliance with NHMRC VTE prevention clinical guidelines [26] and Therapeutic Guidelines Antibiotic [27]. **Supplementary Table 2:** Factors that met criteria for inclusion in regression modelling with Oxford score at 90 days. **Supplementary Table 3:** Factors that met criteria for inclusion in regression modelling with Oxford score at 365 days. **Supplementary Table 4:** Factors that met criteria for inclusion in regression modelling with EQ-56D Index scores at 365 days. **Supplementary Table 5:** Factors that met criteria for inclusion in regression modelling with EQ-56D Index scores at 365 days.

## Data Availability

A deidentified version of the data set and the full R code for all analyses are available in the Australian Research Data Commons (https://doi.org/10.26190/c46r-ne05).

## Abbreviations

AIC: Akaike information criterion
AOR: Adjusted odds ratio
ASA: American Society of Anesthesiology
BIC: Bayesian information criterion
BMI: Body mass index
CPG: Clinical practice guidelines
DVT: Deep vein thrombosis
GCS: Graduated compression stockings
GORD: Gastro-intestinal reflux disorder
ICD: Intermittent compression devices
ICP: Intermittent compression pumps (type of ICD)
IQR: Inter-quartile range
LOS: Length of stay
LTFU: Lost to follow up
LMWH: Low molecular weight heparins
MCID: Various minimal clinically important difference
MICE: Multivariate imputation by chained equations
MRSA: Methicillin-resistant staphylococcus aureus
NHMRC: National Health and Medical Research Council
NSAID: Non-steroidal anti-inflammatories
PE: Pulmonary embolism
PROMs: Patient-reported outcome measures
QALY: Quality-adjusted life years
SCD: Sequential compressive devices
SD: Standard deviation
SE: Standard error
SSI: Surgical site infection
TG: Therapeutic guidelines
THA: Total hip arthroplasty
TKA: Total knee arthroplasty
VAS: Visual analogue scale
VTE: Venous-thromboembolism

## References

[1] Ramkumar PN, Harris JD, Noble PC (2015). Patient-reported outcome measures after total knee arthroplasty: a systematic review. Bone Joint Res.

[2] Shan L, Shan B, Graham D, Saxena A (2014). Total hip replacement: a systematic review and meta-analysis on mid-term quality of life. Osteoarthr.

[3] Shan L, Shan B, Suzuki A, Nouh F, Saxena A (2015). Intermediate and long-term quality of life after total knee replacement: a systematic review and meta-analysis. J Bone Joint Surg.

[4] Kim SM, Moon YW, Lim SJ, Kim DW, Park YS (2016). Effect of oral factor Xa inhibitor and low-molecular-weight heparin on surgical complications following total hip arthroplasty. Thromb Haemost.

[5] MacLean C (2017). Value-based purchasing for osteoarthritis and total knee arthroplasty: What role for patient-reported outcomes?. J Am Acad Orthop Surg.

[6] Kim K, Iorio R (2017). The 5 clinical pillars of value for total joint arthroplasty in a bundled payment paradigm. J Arthroplasty.

[7] Marshall DA, Jin X, Pittman LB, Smith CJ (2021). The use of patient-reported outcome measures in hip and knee arthroplasty in Alberta. J Patient Rep Outcomes.

[8] Churruca K, Pomare C, Ellis LA, Long JC, Henderson SB, Murphy LED, Leahy CJ, Braithwaite J (2021). Patient-reported outcome measures (PROMs): a review of generic and condition-specific measures and a discussion of trends and issues. Health Expect.

[9] Dawson J, Fitzpatrick R, Carr A, Murray D (1996). Questionnaire on the perceptions of patients about total hip replacement. J Bone Joint Surg Br.

[10] Dawson J, Fitzpatrick R, Murray D, Carr A (1998). Questionnaire on the perceptions of patients about total knee replacement. J Bone Joint Surg Br.

[11] Euroqol Research Foundation (2021) EQ-5D-5L user guide, Version 3.0. Rotterdam (NL)

[12] Harris K, Dawson J, Gibbons E, Lim C, Beard D, Fitzpatrick R, Price A (2016). Systematic review of measurement properties of patient-reported outcome measures used in patients undergoing hip and knee arthroplasty. Patient Relat Outcome Meas.

[13] Shim J, Hamilton DF (2019). Comparative responsiveness of the PROMIS-10 global health and EQ-5D questionnaires in patients undergoing total knee arthroplasty. Bone Joint J.

[14] Da Silva RR, Santos AAM, De SampaioCarvalhoJúnior J, Matos MA (2014). Quality of life after total knee arthroplasty: systematic review. Rev Bras Ortop (Sao Paulo).

[15] Okafor L, Chen AF (2019). Patient satisfaction and total hip arthroplasty: a review. Arthroplasty.

[16] Khatib Y, Badge H, Xuan W, Naylor JM, Harris IA (2020). Patient satisfaction and perception of success after total knee arthroplasty are more strongly associated with patient factors and complications than surgical or anaesthetic factors. Knee Surg Sports Traumatol Arthrosc.

[17] Vajapey SP, Mckeon JF, Krueger CA, Spitzer AI (2021). Outcomes of total joint arthroplasty in patients with depression: a systematic review. J Clin Orthop Trauma.

[18] Kahlenberg CA, Nwachukwu BU, Mclawhorn AS, Cross MB, Cornell CN, Padgett DE (2018). Patient satisfaction after total knee replacement: a systematic review. HSS J.

[19] Ghanima W, Wik HS, Tavoly M, Enden T, Jelsness-Jørgensen LP (2018). Late consequences of venous thromboembolism: measuring quality of life after deep vein thrombosis and pulmonary embolism. Thromb Res.

[20] Utne KK, Tavoly M, Wik HS, Jelsness-Jørgensen LP, Holst R, Sandset PM, Ghanima W (2016). Health-related quality of life after deep vein thrombosis. Springerplus.

[21] Carpenter CVE, Wylde V, Moore AJ, Sayers A, Blom AW, Whitehouse MR (2020). Perceived occurrence of an adverse event affects patient-reported outcomes after total hip replacement. BMC Musculoskelet Disord.

[22] Chandrananth J, Rabinovich A, Karahalios A, Guy S, Tran P (2016). Impact of adherence to local antibiotic prophylaxis guidelines on infection outcome after total hip or knee arthroplasty. J Hosp Infect.

[23] Young B, Ng TM, Teng C, Ang B, Tai HY, Lye DC (2011). Nonconcordance with surgical site infection prevention guidelines and rates of surgical site infections for general surgical, neurological, and orthopedic procedures. Antimicrob Agents Chemother.

[24] Clinicaltrials.Gov. (2013) Clinical trial registration [Online]. Bethesda (MD): National Library of Medicine (US). https://www.clinicaltrials.gov/ct2/show/NCT01899443?term=Improving+Services+and+Outcomes+for+Joint+Replacement+Patients&draw=2&rank=1 Accessed 30 June 2019.

[25] Arthroplasty Clinical Outcomes Registry National (2015) Arthroplasty Clinical Outcomes Registry National 2014 annual report Liverpool (AU)

[26] National Health and Medical Research Council (2009) Clinical practice guideline for the prevention of venous thromboembolism (deep vein thrombosis and pulmonary embolism) in patients admitted to Australian hospitals. Melbourne (AU), National Health and Medical Research Council

[27] Therapeutic Guidelines Limited (2010) Therapeutic guidelines antibiotic. Melbourne (AU), Therapeutic Guidelines Limited

[28] R Core Team (2019) R: a language and environment for statistical computing. Vienna (AT), R Foundation for Statistical Computing

[29] Gagnier JJ, Huang H, Mullins M, Marinac-Dabić D, Ghambaryan A, Eloff B, Mirza F, Bayona M (2018). Measurement properties of patient-reported outcome measures used in patients undergoing total hip arthroplasty: a systematic review. JBJS Rev.

[30] Gagnier JJ, Mullins M, Huang H, Marinac-Dabic D, Ghambaryan A, Eloff B, Mirza F, Bayona M (2017). A systematic review of measurement properties of patient-reported outcome measures used in patients undergoing total knee arthroplasty. J Arthroplasty.

[31] King MT (2011). A point of minimal important difference (MID): a critique of terminology and methods. Expert Rev Pharmacoecon Outcomes Res.

[32] Beard DJ, Harris K, Dawson J, Doll H, Murray DW, Carr AJ, Price AJ (2015). Meaningful changes for the Oxford hip and knee scores after joint replacement surgery. J Clin Epidemiol.

[33] Conner-Spady BL, Marshall DA, Bohm E, Dunbar MJ, Loucks L, Al Khudairy A, Noseworthy TW (2015). Reliability and validity of the EQ-5D-5L compared to the EQ-5D-3L in patients with osteoarthritis referred for hip and knee replacement. Qual Life Res.

[34] Ingelsrud LH, Roos EM, Terluin B, Gromov K, Husted H, Troelsen A (2018). Minimal important change values for the Oxford knee score and the forgotten joint score at 1 year after total knee replacement. Acta Orthop.

[35] Hamilton DF, Loth FL, Macdonald DJ, Giesinger K, Patton JT, Simpson AH, Howie CR, Giesinger JM (2018). Treatment success following joint arthroplasty: defining thresholds for the Oxford hip and knee scores. J Arthroplasty.

[36] Browne JP, Bastaki H, Dawson J (2013). What is the optimal time point to assess patient-reported recovery after hip and knee replacement? A systematic review and analysis of routinely reported outcome data from the English patient-reported outcome measures programme. Health Qual Life Outcomes.

[37] Abdel Messih M, Naylor JM, Descallar J, Manickam A, Mittal R, Harris IA (2014). Mail versus telephone administration of the Oxford knee and hip scores. J Arthroplasty.

[38] Buchholz I, Janssen MF, Kohlmann T, Feng Y-S (2018). A systematic review of studies comparing the measurement properties of the three-level and five-level versions of the EQ-5D. Pharmacoeconomics.

[39] Ernstsson O, Janssen MF, Heintz E (2020). Collection and use of EQ-5D for follow-up, decision-making, and quality improvement in health care - the case of the Swedish national quality registries. J Patient Rep Outcomes.

[40] Chatterji R, Naylor JM, Harris IA, Armstrong E, Davidson E, Ekmejian R, Descallar J (2017). An equivalence study: are patient-completed and telephone interview equivalent modes of administration for the EuroQol survey?. Health Qual Life Outcomes.

[41] Bilbao A, García-Pérez L, Arenaza JC, García I, Ariza-Cardiel G, Trujillo-Martín E, Forjaz MJ, Martín-Fernández J (2018). Psychometric properties of the EQ-5D-5L in patients with hip or knee osteoarthritis: reliability, validity and responsiveness. Qual Life Res.

[42] Payakachat N, Ali MM, Tilford JM (2015). Can the EQ-5D tetect meaningful change? A systematic review. Pharmacoeconomics.

[43] Mayhew D, Mendonca V, Murthy BVS (2019). A review of ASA physical status – historical perspectives and modern developments. Anaesthesia.

[44] Alamanda VK, Springer BD (2019). The prevention of infection: 12 modifiable risk factors. Bone Joint J.

[45] Zhang J, Chen Z, Zheng J, Breusch SJ, Tian J (2015). Risk factors for venous thromboembolism after total hip and total knee arthroplasty: a meta-analysis. Arch Orthop Trauma Surg.

[46] Schwartz FH, Lange J (2017). Factors that affect outcome following total joint arthroplasty: a review of the recent literature. Curr Rev Musculoskelet Med.

[47] Harris I, Cuthbert A, Lorimer M, De Steiger R, Lewis P, Graves SE (2019). Outcomes of hip and knee replacement surgery in private and public hospitals in Australia. ANZ J Surg.

[48] Payakachat N, Ali MM, Tilford JM (2015). Can the EQ-5D detect meaningful change? A systematic review. Pharmacoeconomics.

[49] Kawai T, Goto K, Kuroda Y, Matsuda S (2020). Lower activity and function scores are associated with a higher risk of preoperative deep venous thrombosis in patients undergoing total hip arthroplasty. J Clin Med.

[50] Etxeandia-Ikobaltzeta I, Zhang Y, Brundisini F, Florez ID, Wiercioch W, Nieuwlaat R, Begum H, Cuello CA, Roldan Y, Chen R, Ding C, Morgan RL, Riva JJ, Zhang Y, Charide R, Agarwal A, Balduzzi S, Morgano GP, Yepes-Nuñez JJ, Rehman Y, Neumann I, Schwab N, Baldeh T, Braun C, Rodríguez MF, Schünemann HJ (2020). Patient values and preferences regarding VTE disease: a systematic review to inform American Society of Hematology guidelines. Blood Adv.

[51] Calabro L, Clement ND, Macdonald D, Patton JT, Howie CR, Burnett R (2021). Venous thromboembolism after total knee arthroplasty is associated with a worse functional outcome at one year. Bone Joint J.

[52] Trocio J, Rosen VM, Gupta A, Dina O, Vo L, Hlavacek P, Rosenblatt L (2019). Systematic literature review of treatment patterns for venous thromboembolism patients during transitions from inpatient to post-discharge settings. Clin Outcomes Res.

[53] Mansukhani SG, Maclean EA, Manzey LL, Possidente CJ, Cappelleri JC, Deal LS (2021). Development of a new patient-reported medication adherence instrument: concerns influencing medication adherence. Patient Prefer Adherence.

[54] Ritschl V, Stamm TA, Aletaha D, Bijlsma JWJ, Böhm P, Dragoi R, Dures E, Estévez-López F, Gossec L, Iagnocco A, Negrón JB, Nudel M, Marques A, Moholt E, Skrubbeltrang C, Van Den Bemt B, Viktil K, Voshaar M, Carmona L, De Thurah A (2020). Prevention, screening, assessing and managing of non-adherent behaviour in people with rheumatic and musculoskeletal diseases: systematic reviews informing the 2020 EULAR points to consider. RMD Open.

[55] Foley L, Larkin J, Lombard-Vance R, Murphy AW, Hynes L, Galvin E, Molloy GJ (2021). Prevalence and predictors of medication non-adherence among people living with multimorbidity: a systematic review and meta-analysis. BMJ Open.

[56] Wilke T, Muller S (2010). Nonadherence in outpatient thromboprophylaxis after major orthopedic surgery: a systematic review. Expert Rev Pharmacoeconomics Outcomes Res.

[57] Tanner J, Padley W, Davey S, Murphy K, Brown B (2013). Patient narratives of surgical site infection: implications for practice. J Hosp Infect.

[58] Afzal SK, Hasan SS, Babar ZU-D (2019). A systematic review of patient-reported outcomes associated with the use of direct-acting oral anticoagulants. Br J Clin Pharmacol.

[59] Apenteng PN, Fitzmaurice D, Litchfield I, Harrison S, Heneghan C, Ward A, Greenfield S (2016). Patients' perceptions and experiences of the prevention of hospital-acquired thrombosis: a qualitative study. BMJ Open.

[60] Moreno JP, Bautista M, Castro J, Bonilla G, Llinás A (2020). Extended thromboprophylaxis for hip or knee arthroplasty. Does the administration route and dosage regimen affect adherence? A cohort study. Int Orthop.

[61] Arcelus JI, Felicissimo P (2013). Venous thromboprophylaxis duration and adherence to international guidelines in patients undergoing major orthopaedic surgery: results of the international, longitudinal, observational DEIMOS registry. Thromb Res.

[62] Schwartz AJ, Bozic KJ, Etzioni DA (2019). Value-based total hip and knee arthroplasty: a framework for understanding the literature. J Am Acad Orthop Surg.

[63] Pinney SJ, Page AE, Jevsevar DS, Bozic KJ (2015). Current concept review: quality and process improvement in orthopedics. Orthop Res Rev.

[64] Mukherjee P, Khadra M, Merrett N, Rawstron E, Richardson A, Sutherland K, Levesque J-F (2022). Value-based care in surgery: implications in crisis and beyond. ANZ J Surg.

[65] Harris K, Lim CR, Dawson J, Fitzpatrick R, Beard DJ, Price AJ (2017). The Oxford knee score and its subscales do not exhibit a ceiling or a floor effect in knee arthroplasty patients: an analysis of the National Health Service PROMs data set. Knee Surg Sports Traumatol Arthrosc.

[66] Van Hout B, Janssen MF, Feng YS, Kohlmann T, Busschbach J, Golicki D, Lloyd A, Scalone L, Kind P, Pickard AS (2012). Interim scoring for the EQ-5D-5L: mapping the EQ-5D-5L to EQ-5D-3L value sets. Value Health.

[67] Bjorgul K, Novicoff WM, Saleh KJ (2010). Evaluating comorbidities in total hip and knee arthroplasty: available instruments. J Orthop Trauma.

[68] Hunter DJ, Bierma-Zeinstra S (2019). Osteoarthritis. Lancet.

[69] Heath EL, Ackerman IN, Cashman K, Lorimer M, Graves SE, Harris IA (2021). Patient-reported outcomes after hip and knee arthroplasty: results from a large national registry. Bone Jt Open.

[70] Gunaratne R, Pratt DN, Banda J, Fick DP, Khan RJK, Robertson BW (2017). Patient dissatisfaction following total knee arthroplasty: a systematic review of the literature. J Arthroplasty.

[71] Peh KQE, Kwan YH, Goh H, Ramchandani H, Phang JK, Lim ZY, Loh DHF, Østbye T, Blalock DV, Yoon S, Bosworth HB, Low LL, Thumboo J (2021). An adaptable framework for factors contributing to medication adherence: results from a systematic review of 102 conceptual frameworks. J Gen Intern Med.

[72] Malik AT, Jain N, Scharschmidt TJ, Li M, Glassman AH, Khan SN (2018). Does surgeon volume affect outcomes following primary total hip arthroplasty? A systematic review. J Arthroplasty.

[73] Eibich P, Dakin HA, Price AJ, Beard D, Arden NK, Gray AM (2018). Associations between preoperative Oxford hip and knee scores and costs and quality of life of patients undergoing primary total joint replacement in the NHS England: an observational study. BMJ Open.

