# Supplementary material for: Association between VTE and antibiotic prophylaxis guideline compliance and patient-reported outcomes after total hip and knee arthroplasty: an observational study
Source: J Patient Rep Outcomes. 2022 Oct 12;6:110. doi: 10.1186/s41687-022-00502-6 (PMC9556685; doi:10.1186/s41687-022-00502-6)
Supplement: Supplementary file 1 — Additional file 1. Supplementary Table 1: Criteria for compliance with NHMRC VTE prevention clinical guidelines [26] and Therapeutic Guidelines Antibiotic [27]. Supplementary Table 2: Factors that met criteria for inclusion in regression modelling with Oxford score at 90 days. Supplementary Table 3: Factors that met criteria for inclusion in regression modelling with Oxford score at 365 days. Supplementary Table 4: Factors that met criteria for inclusion in regression modelling with EQ-56D Index scores at 365 days. Supplementary Table 5: Factors that met criteria for inclusion in regression modelling with EQ-56D Index scores at 365 days. [file 41687_2022_502_MOESM1_ESM.docx]

Supplementary table 1: Criteria for compliance with NHMRC VTE prevention clinical guidelines [26] and Therapeutic Guidelines Antibiotic [27]

| **Criteria for compliance:** | **VTE prophylaxis** |
| --- | --- |
| 1. Right drug | At least one recommended drug was received: low molecular weight heparins (LMWH), fondaparinux, rivaroxaban or dabigatran etexilate. Warfarin and unfractionated heparin (UFH) were also allowed to enable appropriate patient variations to be considered compliant. The use of non-recommended drugs such as aspirin was ignored in determining compliance. |
| 1. Right dose | Every dose of any recommended drug received must be at a minimum dose based on the recommendations, including at least:   - 40mg enoxaparin sodium (or ≥20mg if renal impairment); - 5000u dalteparin (or ≥2500u if renal impairment), - 2.5mg fondaparinux, - 10mg rivaroxaban, - 150mg dabigatran etexilate, - any dose warfarin or UFH. |
| 1. Right duration of chemical prophylaxis | VTE chemoprophylaxis commences day zero (day or surgery) or by day one and continues for at least 27 days following THA and nine days following TKA, with no more than two missed days for any reason. If a VTE was diagnosed while taking prophylaxis, they were considered compliant for the recommended duration. |
| 1. Right mechanical device/s used | People without any contraindications wore at least one type of mechanical prophylactic device, including foot pumps, calf compressors, or graduated compression stockings (GCS). Only foot pumps or calf compressors were recommended following TKA, but GCS was ignored.  The research team could not agree on measuring the recommended duration of mechanical prophylaxis 'until return to full mobility', so duration was ignored.  Any use or non-use of mechanical prophylaxis was considered compliant for people with contraindications for chemical and mechanical prophylaxis. |
| VTE compliance | Compliant with all four elements (1, 2, 3, and 4) |
| **Criteria for compliance:** | **Antibiotic Prophylaxis** |
| 1. Right drug | Cefazolin or flucloxacillin were recommended. Vancomycin could be added for people who had a history of antibiotic-resistant infection or hospital admission for at least five days within the previous three months. Vancomycin was recommended as a sole agent for people with allergies to cephalosporins, penicillins, or all beta-lactam antibiotics.  The use of gentamycin was ignored for compliance as it was unclear if it was being used for prophylaxis against SSI or indwelling catheter use or removal. |
| 1. Right dose | Received 1g cefazolin or 2g if over 80 kilos; 2g flucloxacillin; 25mg/kg up to 1.5g (≥ 60kg = 1.5g) vancomycin. An intraoperative dose was required for people who had received cefazolin or flucloxacillin and whose surgery was continuing longer than three hours or four hours from the first antibiotic dose. |
| 1. Right pre-op timing | Antibiotic prophylaxis commenced any time before skin incision. The recommendation that antibiotics could start five minutes prior to or just after the release of a tourniquet was ignored for compliance calculations. |
| 1. Right duration | The duration of prophylactic antibiotics was less than 27 hours, and only a single dose of vancomycin was used. |
| Antibiotic compliance | Compliant with all four elements (1, 2, 3, and 4) |

*Indications: allergy to penicillin, cephalosporins or all beta-lactam antibiotics, history of multi-resistant organisms, hospital admission longer than five days within three months preoperatively.

Supplementary Table 2: Factors that met criteria for inclusion in regression modelling with Oxford score at 90 days

| Non-compliance NHMRC VTE clinical guidelines |
| --- |
| Non-compliance TG Antibiotic clinical guidelines |
| Joint |
| Insurance sector (public or private hospital) |
| Sex |
| Baseline EQ-5D VAS score |
| Baseline EQ-5D Index score |
| Oxford Score at baseline |
| Current smoker |
| BMI |
| Education |
| ASA score |
| Comorbid depression |
| Comorbid hypertension |
| Comorbid diabetes |
| Comorbid heart disease |
| Comorbid lung disease |
| Comorbid neurological condition |
| Comorbid liver disease |
| Any other comorbid musculoskeletal condition (type not specified) |
| Comorbid gastro-intestinal reflux disorder (GORD) |
| Comorbid sleep apnoea |
| Other comorbid condition (not specified) |
| History of stroke |
| History of previous VTE |
| Took an antidepressant or anticonvulsant medication for pain preoperatively |
| Took an opioid medication for pain preoperatively |
| Took a non-steroidal anti-inflammatory medication for pain preoperatively |
| First mobilised day 0 or 1 |
| Received routine doppler ultrasound in acute hospital |
| Received blood transfusion |
| Use of cement fixation |
| Previous THA |
| Previous TKA |
| Surgical duration |
| Bilateral THA/TKA |
| Neuraxial anaesthesia |

Supplementary Table 3: Factors that met criteria for inclusion in regression modelling with Oxford score at 365 days

| Non-compliance NHMRC VTE clinical guidelines |
| --- |
| Non-compliance TG Antibiotic clinical guidelines |
| Joint |
| Baseline EQ-5D VAS score |
| EQ-5D baseline Index score |
| Oxford Score at baseline |
| Insurance sector (public or private hospital) |
| Sex |
| Education |
| BMI |
| Current smoker |
| ASA score |
| Comorbid depression |
| Comorbid hypertension |
| Comorbid diabetes |
| Comorbid sleep apnoea |
| Comorbid GORD |
| Comorbid heart disease |
| Comorbid lung disease |
| Comorbid neurological condition |
| History of previous VTE |
| Any other comorbid musculoskeletal condition (type not specified) |
| Previous THA |
| Previous TKA |
| Took an antidepressant or anticonvulsant medication for pain preoperatively |
| Took an opioid medication for pain preoperatively |
| Took a non-steroidal anti-inflammatory medication for pain preoperatively |
| Received routine doppler ultrasound in acute hospital |
| First mobilised day 0 or 1 |
| Received blood transfusion |
| Use of cement fixation |
| Surgical duration |

Supplementary Table 4: Factors that met criteria for inclusion in regression modelling with EQ-56D Index scores at 365 days

| Non-compliance NHMRC VTE clinical guidelines |
| --- |
| Non-compliance TG Antibiotic clinical guidelines |
| Joint |
| EQ-5D Baseline Index score |
| EQ-5D Baseline VAS score |
| Oxford Score at baseline |
| Insurance sector (public or private hospital) |
| Age |
| Sex |
| Education |
| BMI |
| Current smoker |
| ASA score |
| Comorbid respiratory disease |
| Comorbid GORD |
| History of stroke |
| Comorbid depression or anxiety |
| Comorbid musculoskeletal condition (type not specified) |
| Comorbid sleep apnoea |
| Comorbid neurological condition |
| Comorbid liver disease |
| Comorbid diabetes |
| History of previous VTE |
| Took an antidepressant or anticonvulsant medication for pain preoperatively |
| Took an opioid medication for pain preoperatively |
| Took any medication for pain preoperatively |
| First mobilised day 0 or 1 |
| Use of surgical drain |
| Use of cement fixation |
| Surgical duration |

Supplementary Table 5: Factors that met criteria for inclusion in regression modelling with EQ-56D Index scores at 365 days

| Non-compliance NHMRC VTE clinical guidelines |
| --- |
| Non-compliance TG Antibiotic clinical guidelines |
| Joint |
| EQ-5D Baseline Index score |
| EQ-5D Baseline VAS score |
| Oxford Score at baseline |
| Insurance sector (public or private hospital) |
| Education |
| Current smoker |
| ASA score |
| Comorbid respiratory disease |
| Other comorbid disease (not specified) |
| History of stroke |
| Comorbid depression or anxiety |
| Comorbid musculoskeletal condition (type not specified) |
| Comorbid sleep apnoea |
| Comorbid bleeding condition |
| Comorbid cancer (any type) |
| Comorbid hypercholesterolemia |
| Comorbid GORD |
| Comorbid diabetes |
| Comorbid hypertension |
| Previous THA |
| Previous TKA |
| History of previous VTE |
| Took an antidepressant or anticonvulsant medication for pain preoperatively |
| Took an opioid medication for pain preoperatively |
| Took any medication for pain preoperatively |
| First mobilised day 0 or 1 |
| Bilateral THA or TKA |
| Use of any neuraxial anaesthesia |
| Use of surgical drain |
| Use of cement fixation |
| Surgical duration |
